# Impact of Polylactic Acid Fibers in Cellulose Nonwoven Mulch Blends on Biodegradability and Performance—An Open Field Study

**DOI:** 10.3390/polym16020222

**Published:** 2024-01-12

**Authors:** Dragana Kopitar, Paula Marasovic, Domagoj Vrsaljko

**Affiliations:** 1 Department of Textile Design and Management, Faculty of Textile Technology, University of Zagreb, Prilaz Baruna Filipovica 28a, 10000 Zagreb, Croatia; paula.marasovic@ttf.unizg.hr; 2 Department of Thermodynamics, Mechanical Engineering and Energy, Faculty of Chemical Engineering and Technology, University of Zagreb, Trg Marka Marulica 19, 10000 Zagreb, Croatia; dvrsal@fkit.unizg.hr

**Keywords:** cellulose fibers, PLA fibers, nonwoven mulch blends, biodegradability, FTIR, soil temperature and moisture, weed control

## Abstract

The performance and degradation of nonwoven mulches made from viscose, jute, hemp fibers, and their blends with PLA fibers, subjected to field conditions, are investigated. This research explores the possible substitution of traditional agricultural polyethylene mulching agro foil with environmentally friendly biodegradable nonwoven mulches produced from blends of jute, hemp, and viscose fibers along with PLA fibers. The nonwoven mulches underwent a ten-month exposure to field conditions, showing varied degradation. The jute and hemp nonwoven mulches degraded completely within the test period, whereas their blends with PLA fibers exhibited slowed degradation. This study indicated that PLA fibers in blends with jute, hemp, and viscose mulches slowed degradation, impacting their structural integrity and tensile properties. The tensile properties of nonwoven mulches blended with 20% of PLA fibers increased the breaking forces after field exposure. Observations on structural changes through microscopy highlighted the structure maintenance in jute and hemp blends due to the non-degraded PLA fibers, contrasting the complete degradation of 100% jute and hemp mulches. A microscopic analysis revealed alterations in the fiber structure and density changes, particularly in viscose mulches and their blends with PLA fibers. Soil temperature variations were observed under different mulches; e.g., agro foil consistently exhibited higher temperatures compared to nonwoven mulches. Notably, the hemp and jute/PLA blend mulches showed slightly elevated temperatures, while the viscose-based mulches consistently revealed the lowest temperatures. Regarding soil moisture, the nonwoven mulches generally maintained higher moisture levels compared to the control field and agro foil from June to October. These findings suggest that nonwoven mulches effectively preserved soil moisture during critical growth periods, potentially positively impacting plant growth. The weed suppression capabilities varied among mulches, with hemp mulch initially displaying the lowest suppression ability in the first six months. The addition of 20% of PLA fibers in mulch blends with viscose, jute, and hemp notably improved the weed control capabilities. Understanding the impacts of field conditions on newly produced nonwoven mulches is crucial for optimizing mulch selection in agricultural practices to enhance soil conditions and weed management.

## 1. Introduction

Blending natural fibers with polylactic acid (PLA) biopolymers in nonwoven mulch production can have many benefits, such as enhanced physical properties, performance optimization, and customization. To meet the specific requirements of different applications or target markets, a blended composition of nonwoven mulches can be tailored. A PLA biopolymer alone may not possess all of the desired physical properties that are required for nonwoven mulch applications. Adding a PLA biopolymer in nonwoven mulch made of natural fibers can help optimize performance in terms of water retention, weed suppression, soil erosion control, or moisture permeability. In addition, by combining PLA with other biopolymers or natural fibers, the resulting composite material can exhibit improved strength, flexibility, tear resistance, or other specific properties needed for its intended application, such as faster or slower biodegradation. Finally, blending PLA with less expensive materials allows for cost reduction to be achieved while maintaining a certain level of performance [1,2,3,4].

The published research on PLA nonwoven fabric blend biodegradation mainly focuses on blending with polyhydroxyalkanoate (PHA) or polyhydroxybutyrate (PHB) biopolymers and production using the spun-bond or melt-blown processes. The study by Dharmalingam et al. tested the degradation time of biodegradable bio-based spun-bond black and white as well as melt-blown nonwoven mulches made of PLA and a blend of PLA/PHA (70/30). The mulches were exposed to compost-enriched soil in a greenhouse for 45 days, where the mulches’ masses per unit area and tensile strengths were measured to obtain conclusions about their biodegradation. The results showed that the microbial activity was more pronounced for the PLA/PHA blend, indicating that microbes started to use the more readily available components of the mulches as carbon sources. In addition, the tensile strength of the blends decreased significantly after 45 days of exposure [5]. 

Habolt et al. investigated different types of mulches, subjecting them to simulated weathering for 21 days. They found that simulated weathering weakened the mechanical strengths and molecular weights of some mulches, promoting biodegradation. Additionally, PHA inclusion in PLA-based nonwoven mulches improved their biodegradability, with the highest rate of biodegradation being found in the melt-blown PLA-PHA-75/25 blend [6].

Liu et al. investigated the accelerated biodegradation of PLA/PHB blended nonwovens in the presence of a microbial community. The nonwovens were buried in natural soil for 56 days, after which soil samples were collected for subsequent bacterial community domestication. The study revealed that the domesticated strains of bacteria in the Gen III community, including Proteobacteria and Firmicutes, accelerated the degradation of PLA/PHB-blended nonwovens compared to natural soil microbial communities. Overall, the study exhibits that identifying the microbial communities involved in enhancing polymer biodegradation and understanding the resulting composition change are necessary to reveal the mechanism promoting the degradation of renewable polymers [3].

It is well known that a PLA biopolymer degrades faster in increased temperature conditions, in a laboratory, and in a field, where degradation is accelerated via UV radiation since it leads to biopolymer photo-degradation [7,8]. When exposing PLA materials to UV radiation, the energy from the photons can break the chemical bonds within the material, leading to structural changes and the degradation of its properties, meaning the polymer chain absorbs a photon, leading to the chain scission of the C-O bonds. The formation of hydroperoxides leads to degradation, and carboxylic acid and diketone end groups are formed. 

Natural fiber exhibits discoloration after weathering mainly due to lignin and extractive degradation. Lignin absorbs between 80 and 95% of the radiation through the α-carbonyl, biphenyl, and ring-conjugated groups that react with oxygen to form chromophoric groups. For this reason, lignin is responsible for absorbing UV radiation and catalyzing the degradation of PLA. Therefore, blending PLA biopolymers with natural fibers can lead to the faster biodegradation of PLA/natural fiber nonwoven fabrics [9]. 

According to the available literature, there is an almost non-existent research area of nonwoven mulch; it is produced on a card, bonded through a needle-punching process, and is made from natural fibers in blends with PLA fibers. We conducted a biodegradation analysis and performance analysis in outdoor conditions. PLA acid blends with natural fibers, such as jute, kenaf, or bamboo, usually come as composites due to their exceptional qualities, such as green degradability, friendly processing, and lightweight characteristics. Newly made jute/PLA composites made from needle-punched nonwoven jute and a PLA membrane in the study by Fang et al. exhibited excellent mechanical properties and slightly lower thermal stability, which made them suitable for various applications, especially considering their low price and the possibility of degradation [10]. 

A similar conclusion regarding the increase in the tensile strength of PLA composites was reached by Plackett et al., in which the tensile strength was approximately doubled by using jute fiber (40 wt.%). They further mentioned that the broader adoption of natural fibers in polymer reinforcement will benefit from the development of global standards and methods for producing agro-fibers that ensure consistent material quality across different seasons and years [11].

During the 10 months of exposure to field conditions, the degradation of needle-punched nonwoven mulches made of jute, hemp, and viscose fibers in blends with PLA fibers was investigated. The effects of nonwoven mulch blends on soil temperature and moisture in relation to 100% nonwoven mulch and weed suppression properties were analyzed. The possibility of replacing conventional mulching agro foil with biodegradable nonwoven mulches has been established.

## 2. Materials and Methods

### 2.1. Materials

The seven nonwoven mulches were produced from the following fibers: hemp (58.54 dtex), jute (31.02 dtex), and viscose (1.78 dtex, 40 mm). These fibers were provided by Derotex, and the blends with PLA fibers were derived from corn starch (6.84 dtex, 64 mm) and supplied by NatureWorks BV, Plymouth, MN, USA.

The mulches were produced on a card and bonded through the needle punch process with the same production parameters. The nominal mulch mass per unit area was 400 g m^−2^. Jute, hemp, and viscose fibers were blended with PLA fibers in a ratio of 80:20.

A commercially available low-density polyethylene agro foil (Gerovit, Serbia; thickness of 20 µm, 28.17 g m^−2^) and a control field (bare soil) were included in the experiment to compare the biodegradation and performance of produced mulches with traditional mulching material and non-mulched soil. 

The nonwoven mulches and agro foil, with dimensions of 1.5 m × 1.5 m (2.25 m^2^), were randomly placed on the soil in blocks of seven replication plots. The control field was included in each block of replication plots. 

The field test was conducted in Donji Laduč, Croatia (45°53′ N, 15°44′ E), which is characterized by a humid continental climate (Dfa) according to Köppen–Geiger’s classification. On 25 April 2022, the experiment started, and the last nonwoven mulch replication plot was collected in February 2023. One replication plot of nonwoven mulches and agro foil were removed after 60, 120, 180, 240, and 300 days of exposure. During the mulch removal process, the weed suppression ratio was determined. 

The soil temperature and moisture beneath the mulches on the field were recorded once per week, where the degradation of mulches was recorded throughout the physical–mechanical tests after each removal from the experimental field.

### 2.2. Methods

Soil temperature and moisture beneath mulches were recorded once per week throughout the 300-day trial period to evaluate the influence of mulches on the soil. Soil moisture was determined using a PMS-714 soil moisture meter at a soil depth of 15 cm. The soil temperature was measured at the same depth with a Fisher brand bi-metal dial thermometer with a long probe made of waterproof stainless steel.

The nonwoven mulches’ biodegradation ratio was evaluated periodically through the physical–mechanical properties of mulches. Mass per unit area was determined according to the ISO 9073-1:2023 standard for nonwoven textiles [12]. Nonwoven mulch thickness was tested according to ISO 9073-2:1995, applying a pressure of 0.5 kPa [13]. The air permeability of nonwoven fabric was measured on the Air Tronic device, Mesdan S.p.A., according to the ISO 9073-15:2007 standard [14]. A flow rate of 10 liters per minute was used, regulating the airflow until desired pressure drops of 50 Pa were achieved, using a circular test area of 10 cm^2^. The nonwoven mulches were analyzed before and after being exposed to the field conditions, using a Dino-Lite Edge microscope equipped with a 5 MP digital camera. The analysis was conducted at a magnification level of ×65. The mulches’ breaking force and elongation were determined according to the ISO 9073-3:2023 standard for nonwoven fabric on wide strips [15]. The five samples per mulch type in the machine (MD) and five in the cross-machine direction (CD), with dimensions of 350 mm × 200 mm, were tested on the Tenso Lab 5000 Mesdan S.p.A tensile tester at a constant speed of 100 mm/min with a pretension of 5 N.

The collected weeds grown through the mulches were dried until they resembled the absolutely dry sample and then weighed on an analytical balance. The mass of weeds on the control field (uncovered field with mulch) was taken as 100% of weeding, where the percentage of weeding on each mulch and agro foil was calculated and expressed regarding the weeding of the control field.

Fourier-transform infrared (FTIR) analyses were performed on both the untreated control mulches and the samples obtained from the mulches exposed to field conditions. These analyses were performed using an FTIR spectrometer (PerkinElmer Spectrum One, MA, USA) at normal room temperature and typical humidity. To ensure the accuracy and reliability of the measurements, the solid samples were placed on the ATR cell on a Zn/Se crystal in their natural state. Special care was taken to ensure that the samples completely covered the surface of the crystal and that uniform pressure was applied. The spectral data were recorded in the 4000 cm^−1^ to 650 cm^−1^ range, with a spectral resolution of 4 cm^−1^, allowing for the precise and detailed investigation of molecular vibrations and chemical properties.

## 3. Results

Mulches made of 100% viscose, jute, hemp, and PLA fibers and their blends with 20% PLA fibers in a nominal mass per unit area of 400 g m^−2^ were produced. The impact of PLA fibers in cellulose nonwoven mulch blends on performance and degradation were compared with 100% cellulose mulches and conventional agro foil in the field experiment. The samples’ labels are presented in Table 1.

### 3.1. Mass per Unit Area, Thickness, and Air Permeability of Nonwoven Mulches

**Figure 1:**
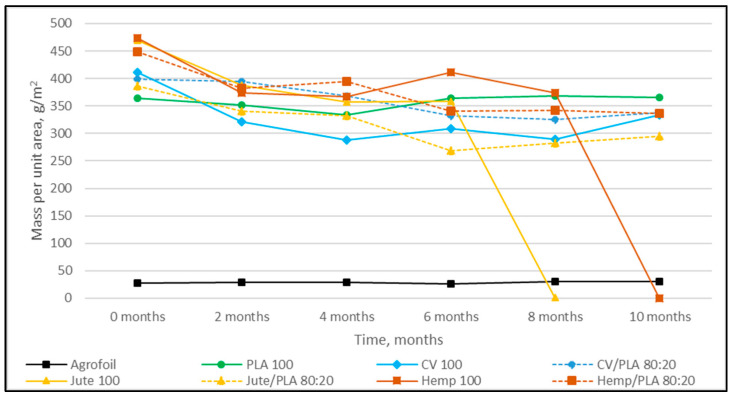
Dependence of nonwoven mulch mass per unit area on exposure time to field conditions.

**Figure 2:**
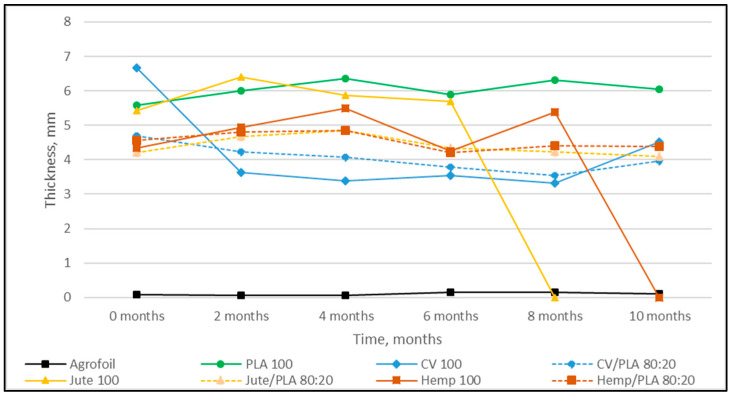
Dependence of nonwoven mulch thickness on exposure time to field conditions.

**Figure 3:**
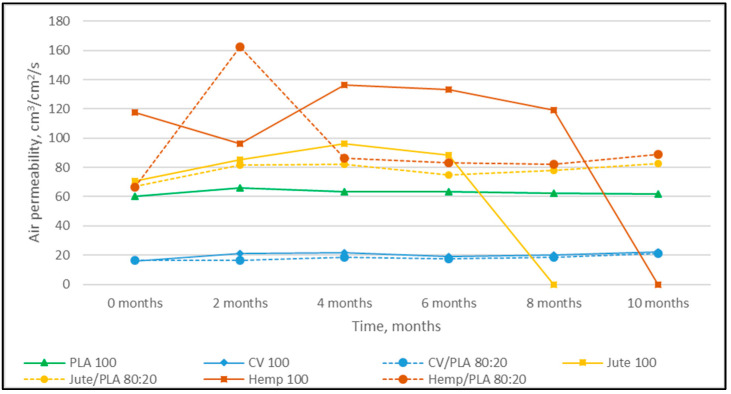
Dependence of nonwoven mulch air permeability on exposure time to field conditions.

The nonwoven mulches’ masses per unit area, thicknesses, and air permeability, before being exposed to field conditions (0 samples) and after 2, 4, 6, 8, and 10 months of exposure, are presented in Figure 1, Figure 2 and Figure 3. 

The mulches produced by 100% jute and hemp fibers degraded during the test period. The jute mulch degraded completely between 6 and 8 months, while the hemp mulch degraded after eight months of exposure. After ten months of exposure to weather conditions, the mass of viscose mulches and all other blends with PLA fibers (nonwoven mulches blends of viscose, jute, and hemp) decreased from 18% to 33% (Table 2). The mulches produced from PLA fibers did not change in mass after ten months of exposure. The agro foil mass per unit area, after ten months of exposure, increased by 5%, probably as the result of soil and residual impurities.

After ten months of exposure to field conditions, the thickness of viscose mulch decreased by 47% (Figure 2). The thickness of mulches made from blends with PLA fibers decreased between 2% and 19%, where the greatest thickness decrease was visible for the viscose/PLA mulches (Table 2). After 10 months of exposure to field conditions, the mulch made of 100% PLA fiber was 8% thicker, while the agro foil was 25% thicker.

The mulches’ air permeability after ten months increased for all tested mulches. The smallest air permeability increase was observed for the PLA mulches, and the largest value was observed for the viscose mulches (Figure 3, Table 2).

The mulches made from stem fibers, jute, and hemp completely decomposed after 10 months, and the jute mulches degraded first. Jute and hemp mulches blended with PLA fibers have approximately equal tendencies in surface mass, thickness, and air permeability decrease, although changes are more pronounced for hemp mulch. Viscose mulches in a blend with PLA fibers show a similar tendency in mass and air permeability reduction to 100% viscose mulches, while the difference in thickness is significant (CV/PLA is 19%; CV 47%). It can be seen that PLA fibers in mulch blends with jute, hemp, and viscose fibers slowed down the losses of surface mass, thickness, and air permeability. The PLA fiber mulches did not show degradation after ten months. 

In general, by observing the properties of all nonwoven mulches over time, it is evident that the properties do not change linearly over time as expected. The reason for this could be due to changes in the structure of mulches during field exposure, i.e., the shrinkage and expansion of fabrics under the influence of the environment (soil, air, water, and plants), field (soil moisture, crusting, temperature, and structure), and microclimate (air temperature, wind speed, air humidity, and solar radiation) conditions [16].

### 3.2. Tensile Properties of Nonwoven Mulches

The breaking force in the cross-machine direction (CD) of all mulches is higher than in the machine direction (MD) as a consequence of the manufacturing process, i.e., the fiber orientation in the nonwoven fabrics produced on the card (Table 3 and Table 4).

The breaking force of mulch blends (20% of PLA fibers) is higher than the breaking force of mulches made of pure fibers in both MD (from 149% to 386%) and CD (203% to 304%) production (Table 3 and Table 4). After two months of exposure, the breaking forces in both directions of almost all mulches increased significantly (MD, 45–406%; CD 56–396%), except for the hemp/PLA mulches blend, where a decrease of 42% in the MD and a decrease of 61% in the CD are evident. 

In the following four months of exposure, a drop in the breaking force in both production directions is recorded for all samples, except for hemp/PLA, which, at the sixth month of exposure, records a significant increase in the breaking force in both directions (MD 197%; CD 187%). A slight increase in the breaking force of PLA mulches is also visible (MD 7%; CD 16%). At the eighth month of exposure, the jute mulches decomposed. The hemp mulches degraded in the 10th month of exposure. After ten months of exposure, viscose and jute mulch blends have a higher breaking force than non-exposed mulches. Only the hemp/PLA blends have a break force drop of 1% in the MD and 44% in the CD. Interestingly, after ten months of exposure, 100% PLA mulches have a significantly higher breaking force in both production directions (MD 267%; CD 323%).

A statistical analysis of the breaking force in the MD and CD was performed. An ANOVA test of the breaking force in the MD and CD showed that there are statistical differences between all mulches, except for the breaking force of nonwoven mulch blends in the MD and CD (Table 5). It can be concluded that the addition of only 20% of PLA fibers into mulches made of cellulose fibers (viscose, hemp, and jute), after ten months of exposure to field conditions, changes the structure in such a way that the breaking force of almost all samples is greater than that before the field exposure. The increase in the breaking force after ten months of field exposure is visible only for mulches made of PLA fibers, which means that mulches made of viscose, jute, and hemp fibers with the addition of only 20% of PLA fibers behave like PLA mulches in terms of tensile properties. There is no statistical difference in the breaking force change of nonwoven fabric blends in the 300 days of exposure to field conditions.

An ANOVA test of the breaking force in the MD and CD of nonwoven mulches and agro foil showed that there are statistical differences between all mulches and agro foil in both production directions. There are significant differences in the degradation of viscose, jute, and hemp mulches, whereas their change in breaking force statistically differs from the degradation of agro foil.

The elongation at break of all tested mulch types is higher in the MD than in the CD (Table 6 and Table 7). Viscose, jute, and hemp mulches blended with 20% PLA fibers have a higher elongation at break than 100% viscose, jute, and hemp mulches in the MD (from 70% to 227%) and CD (124% to 303%). After two months of exposure, the elongation at break of viscose mulch and PLA mulch increased, while the elongation at break of hemp and jute mulch and their blends with PLA fibers visibly decreased. 

The elongation at break of viscose mulch (including viscose blend) and 100% PLA mulch tended to increase in the MD until the end of the exposure time and tended to decrease in the CD. The elongation at break of hemp and jute mulch and their blends with PLA fibers tends to decrease in the MD direction.

In the CD, the mentioned trend is not visible, i.e., the continuous increase and decrease in the elongation at break of jute and hemp mulch blends during the exposure time are recognizable.

Additionally, after ten months of exposure, the elongation at break of the mulches decreased in the MD direction for all mulches, while in the CD direction, the elongation at break for the mulches made of PLA fibers and blends of jute and PLA fibers were slightly higher (10.42% and 6.78%) compared to the control mulches that were not exposed to weathering conditions. The mulches produced from viscose and hemp fibers tend to experience a decrease in the elongation at break in both production directions during the decomposition time. The elongation at break of PLA mulches during exposure increases and decreases without a visible regularity (up to 24% of the absolute value). Blends of viscose, jute, and hemp mulches with PLA fibers have a more pronounced tendency to increase and decrease during exposure time compared with PLA mulches (CV/PLA up to 90%, jute/PLA up to 42%, hemp/PLA up to 193% absolute value). The PLA fibers in the mulch blends visibly affected the elongation at break of the mulches.

A statistical analysis of the elongation at break provides similar conclusions as for the breaking force (Table 8). Accordingly, there are no statistically significant differences in the elongation at break of nonwoven mulch blends in the MD and between nonwoven mulch blends in the MD and agro foil. There are significant differences in the elongation at break of 100% nonwoven mulches and between all nonwoven mulches and agro foil in both production directions. 

It would be expected that the breaking force and elongation of the mulches would decrease during the field exposure period, meaning mulch degradation occurs due to weather conditions. Since the results of mulch degradation were not obtained, additional analyses were performed to gain a better insight into the degradation of the fibers and changes in the mulch structure due to field influences.

A clear trend in the mulch dimensions’ changes during ten months of exposure has not been established (Table 9). Although mulches mostly shrunk after ten months of exposure, during that period, the mulches’ dimensions altered in both production directions, meaning they were shrunk and extended. After ten months of exposure, almost all mulches shrunk, both in the MD and CD, except for the PLA mulch, which was slightly extended (1.0% in MD and 1.5% in CD), and the mulch made of jute/PLA fibers in the MD (slightly extended for 1.5%). Dimensional changes during the exposure period certainly influenced the mass per unit area, thickness, air permeability, and tensile properties that were measured in certain periods.

**Figure 4:**
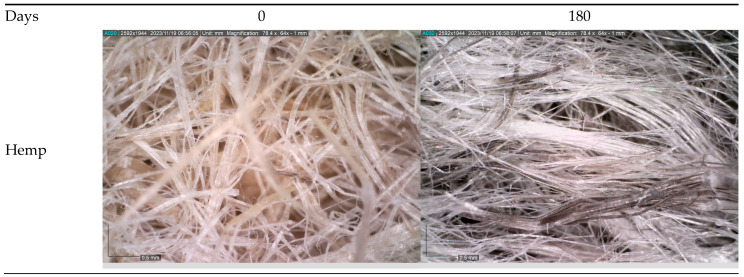

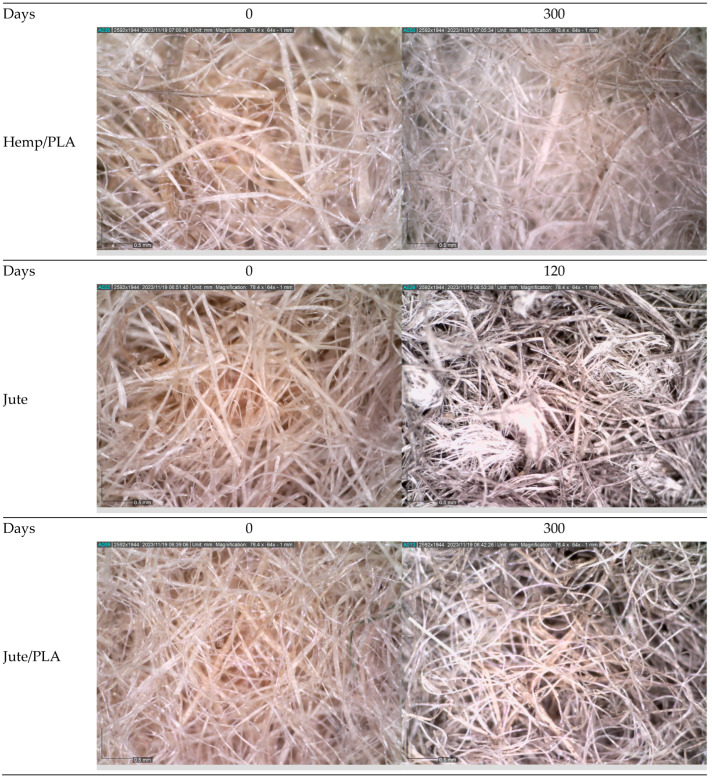

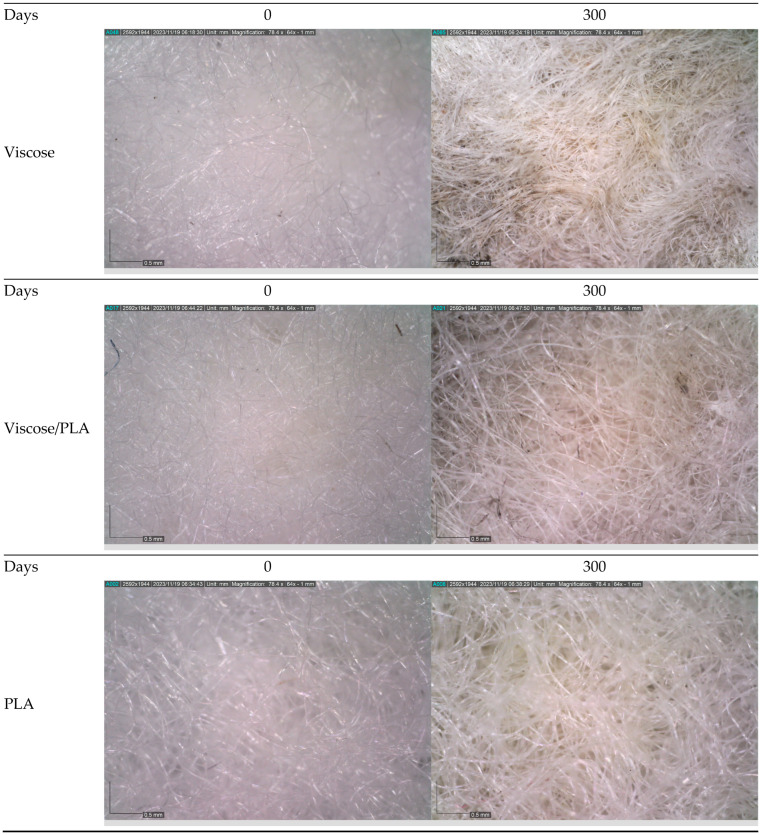
Microscopic images of unexposed nonwoven mulches and nonwoven mulches after ten months of field exposure; magnification ×65.

The microscopic images of the unexposed mulches and mulches after 300 days of field exposure were taken to investigate the structural changes (Figure 4). Also, the density of nonwoven mulches was calculated through ten months of exposure to weather conditions (Table 10). To calculate the change in the nonwoven mulch density due to exposure to field conditions, the nonwoven mulch density was calculated based on the mass per unit area and thickness of the 0 sample and samples after ten months of exposure to weathering. Since jute and hemp nonwoven mulches degrade after eight to ten months, their densities were calculated before they degraded. Nonwoven fabric packing density, or bulk density, is the mass per unit volume of the nonwoven fabric (kg/m^3^). It equals the measured weight per unit area (kg/m^2^) divided by the measured thickness of the fabric (m).

When observing the changes in the structure and density of nonwoven mulches after exposure to field conditions, the difference between mulches produced from different types of fibers is evident.

The jute and hemp mulches degraded to such an extent that it was impossible to collect the mulches from the field, i.e., pieces of the sample remained in the hands. A microscopy of the remains of jute and hemp nonwoven mulches shows the decomposition of jute and hemp fibers into elementary fibers. The calculated reduction in the density of jute mulch before decomposition is 27.0%, and that of hemp is 36.1%.

On the other hand, in the nonwoven mulch blends from jute and hemp with PLA fibers, the maintenance of the structure due to non-degraded PLA fibers is visible. The decrease in the density of nonwoven blends of jute (21.9%) and hemp (21.9%) mulches is less than that of mulches produced from 100% jute (27.0%) and hemp (36.1%) fibers. When considering the calculation of the density and the microscopic pictures of the mulches after ten months of exposure to field conditions, it can be concluded that the non-degraded PLA fibers in the jute and hemp mulch blends contribute to the maintenance of the structure.

The above is additionally supported by the nonwoven mulches’ tensile properties. By comparing the breaking force and elongation at break of nonwoven jute and hemp mulches and their blends with PLA fibers in the MD, it is evident that the breaking force of jute and hemp mulches tend to decrease after the initial growth after two months, while their blends show an increase after six months, followed by a less intensive decline until the end of the exposure period.

In the CD, the breaking force has the same tendency as in the MD, except that in the case of jute blends, an alternating increase and decrease in the breaking force is visible. 

The elongation at break of jute and hemp nonwoven mulches in both directions of production (MD and CD) recorded a decrease, while in the case of the blends, alternating increasing and decreasing trends in the breaking force are visible in both directions of production.

The microscopic images of mulches made of viscose fibers show that the fibers have retained their shape and length but turned yellow due to a certain degree of decomposition. 

A significant change in the structure of the mulches is also visible, i.e., the fibers have come closer to each other, creating a higher density nonwoven structure (the spaces between the fibers filled with air have decreased) compared to the mulch that was not exposed to the field conditions. Due to the change in the structure, there was a significant reduction in the thickness (47%) and a density increase (19.3%) in the viscose nonwoven mulches. For CV/PLA mulch blends, the density increases by only 0.4%, and the thickness reduces by 19%. Also, the microscopic image of the viscose mulch blended with PLA fibers shows the same but less pronounced structural change, i.e., the fibers come closer to each other.

The breaking force of the viscose mulches increased significantly after two months of exposure to the field conditions, followed by a total breaking force decrease of 10% in the MD and 27% in the CD after ten months. 

The CV/PLA nonwoven mulch blend recorded a significant increase in the breaking force after two months, after which alternating increasing and decreasing trends were recorded. After ten months, the breaking force was 180% higher in the MD and 73% higher in the CD. The dimensional changes of mulches produced by viscose fibers and mulches with a blend of CV/PLA fibers are the same, as they reduced by 6.3% in the MD and 0.7% in the CD. 

The addition of PLA fibers in the blend with viscose fibers contributed to the breaking force increase and significantly smaller changes in the elongation at break in both directions after ten months of exposure. 

In addition, it should be considered that the breaking force of PLA mulches in the MD after ten months of exposure was increased by 267%, and in the CD, it was increased by 323%. The reduction in breaking force in the MD was only 9.12%, while in the CD, it increased by 10.42%. The mulches made of PLA fibers changed their dimensions the least, i.e., their dimensions increased by 1% in the MD and by 1.5% in the CD.

Additional research is needed to investigate whether PLA fibers in jute, hemp, and viscose nonwoven mulches affect the degradation rate of the jute, hemp, and viscose fibers themselves.

### 3.3. FTIR Analyses of Mulches through the Field-Testing Period

To determine to what extent the structure of the nonwoven mulch, i.e., the fibers within the mulch structure, influenced the mass per unit area, thickness, air permeability, and tensile properties that should show the degradation of the mulch, an FTIR analysis was carried out.

In the 3600–3200 cm^−1^ range, viscose fibers, primarily made of cellulose, display a distinct band indicating hydroxyl (OH) group stretching vibrations. Bands between 3000 and 2800 cm^−1^ represent stretching vibrations of carbon–hydrogen (CH) bonds in cellulose’s aliphatic side chains, while the 1200–1000 cm^−1^ range reflects CO bond stretching vibrations. Moreover, peaks at 1470–1420 cm^−1^ indicate the bending vibrations of CH bonds in cellulose’s aliphatic side chains. These unique bands not only confirm the presence of cellulose, but also offer specific wavenumbers and intensity information that can indicate changes in viscose fiber degradation when exposed to environmental conditions [17,18]. A reduction in the band intensity at higher wavenumbers (3333 cm^−1^ and 2915 cm^−1^) in exposed viscose fibers suggests alterations or the loss of certain functional groups, leading to modifications in molecular vibrations during degradation. Slight changes are observed at lower wavenumbers (2916 cm^−1^, 2730 cm^−1^, and 2541 cm^−1^), indicating the formation of new functional groups within the fiber structure. Minor changes at lower wavenumbers (below 1000 cm^−1^) may be attributed to signals from other molecules in this range. Of particular interest is the region around 3000 cm^−1^, where alterations in the hydroxyl band are linked to changes in the crystalline structure, indicating fiber degradation. 

**Figure 5:**
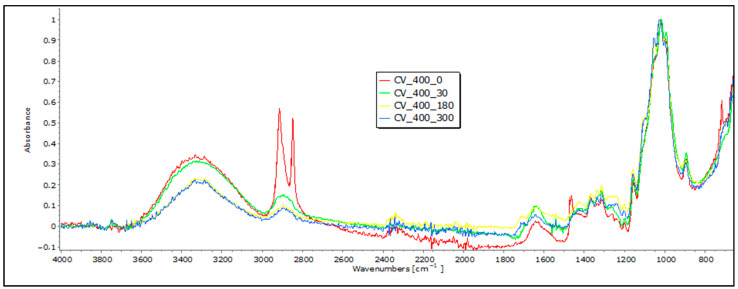
FTIR spectra of viscose (CV) nonwoven mulches of 400 g m^−2^ exposed for 0, 30, 180, and 300 days.

The analysis of viscose fibers from nonwoven mulches reveals a characteristic band at 2915 cm^−1^, which remains unchanged after 30 days of exposure (Figure 5). However, prolonged exposure causes the band to change slightly, indicating a molecular structure change. Two additional bands at 2848 cm^−1^ and 2730 cm^−1^ are present in the 0 sample but disappear in the exposed fibers, indicating significant changes in molecular vibrations or functional groups. Further insight into the structural alterations comes from shifts in the band positions in the region between 1800 and 600 cm^−1^. These shifts, attributed to variations in the hydrogen bonding scheme and changes in dihedral angles at the glycosidic linkage, reflect modifications in the bending and stretching vibrations of molecular bonds in cellulose fibers [17]. Additionally, bands around 1700–1500 cm^−1^, corresponding to carbonyl groups and absorbed water, indicate the collapse of the cellulose structure over time, accompanied by an increase in water molecule absorption [19,20]. In a viscose FTIR analysis, the appearance of additional peaks at 1720 cm^−1^, observed at 180 and 300 days, indicates significant structural changes. Shifts in the 1800 to 600 cm^−1^ range suggest variations in hydrogen bonding and glycosidic linkage angles. The changes in the bending and stretching vibrations of molecular bonds in cellulose fibers, coupled with shifts around 1700–1500 cm^−1^, signify the degradation process. Specifically, the 1720 cm^−1^ peak highlights crucial alterations in the cellulose structure over time. Together, these FTIR spectral changes provide compelling evidence of the chemical transformations occurring in viscose fibers during the degradation process, shedding light on the dynamic evolution of their molecular and structural characteristics.

Cellulose serves as the primary structural element in bast fibers, such as hemp and jute, and it is accompanied by a smaller quantity of hemicellulose and various other non-cellulosic constituents, which include lignin, pectin, fats, waxes, water, pigments, minerals, and ashes, which are present in differing proportions. In the FTIR spectra of these bast fibers, we observe characteristic bands associated with cellulose, including OH stretching (around 3600–3300 cm^−1^), CH stretching (around 2900 cm^−1^), and CH_2_ bending (around 1200 cm^−1^) [21,22,23]. These FTIR profiles for hemp and jute fibers typically reflect distinctive chemical compositions and molecular structures, with band positions and intensities varying depending on factors like cultivar, agro-ecological conditions, fiber extraction, mechanical processing, and differences in the chemical composition and structure. It is crucial to note that bast fibers may also contain hemicellulose and lignin, potentially introducing additional bands in the FTIR spectra. The presence and intensity of these bands depend on the amount of pectin and lignin that is present. An interpretation of the characteristic bands of lignin and pectin requires care, as they may closely overlap. Lignin’s characteristic bands are typically found in the 1700–1600 cm^−1^ range, signifying aromatic ring stretching vibrations, and at 900–830 cm^−1^, indicating CH aromatic bending vibrations [21]. On the other hand, pectin’s bands may involve OH stretching in the 3600–3200 cm^−1^ range and CO stretching in the 1200–1000 cm^−1^ range. To differentiate between these compounds, it is essential to identify the specific functional groups associated with each band. For instance, lignin contains aromatic rings, leading to peaks related to aromatic CC bonds, whereas pectin contains carboxyl groups (COOH), and potentially yields bands in the 1750–1700 cm^−1^ range. Lignin’s aromatic ring stretching bands are typically more intense and prominent compared to pectin’s bands [20].

**Figure 6:**
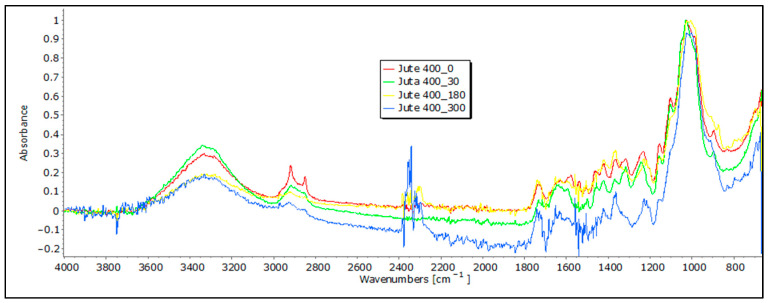
FTIR spectra of jute nonwoven mulches of 400 g m^−2^ exposed for 0, 30, 180, and 300 days.

**Figure 7:**
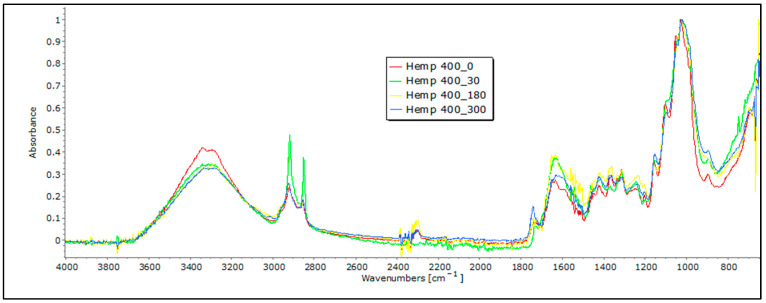
FTIR spectra of hemp nonwoven mulches of 400 g m^−2^ exposed for 0, 30, 180, and 300 days.

Following exposure to field conditions, the FTIR spectra of jute and hemp fibers display significant changes in characteristic band positions and reduced band intensities, which are indicative of degradation processes and structural alterations, often attributed to the breakdown and loss of cellulose chains (Figure 6 and Figure 7). 

In the case of hemp and jute, a decrease in intensity is visible in certain bands. For example, a decrease in characteristic bands associated with cellulose, including OH stretching, is visible, concerning the 0 sample. The intensity of the band decreases in samples that were exposed to external influences, which could suggest a move from highly crystalline to amorphous cellulose, which means there is an ongoing degradation of the material [20]. The most significant decrease in band intensity is evident in the FTIR spectrum of jute fibers after 300 days of exposure to field conditions, particularly at the 1030 cm^−1^ and 1745 cm^−1^ characteristic bands associated with pectin and lignin. This decrease could signify a reduction in the concentration of these chemical components [21,22,23]. Also, it is important to note that the appearance of bands around 1700 cm^−1^ can indicate the presence of some impurities in the initial fibers, such as fats, waxes, and resins, which are particularly visible with hemp samples exposed to external influences for a longer time [20]. In addition, the materials that were tested had some impurities on them caused by exposure to soil, which the FTIR analysis picked up and presented as an increase in some bands, so any conclusions on the degradation of nonwoven material should be confirmed using other methods.

**Figure 8:**
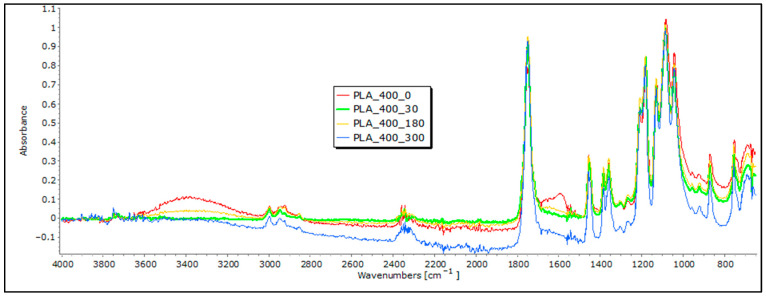
FTIR spectra of PLA nonwoven mulches of 400 g m^−2^ exposed for 0, 30, 180, and 300 days.

In the FTIR analysis of the nonwoven mulch made from PLA fibers before exposure, distinct bands were identified (Figure 8). These included a band around 3400 cm^−1^, representing the stretching vibration of hydroxyl groups (OH), and a band at 1750 cm^−1^, signifying the stretching vibration of the carbonyl group (CO) within the ester linkage of PLA. Additionally, there are bands around 2997 cm^−1^, which are associated with the stretching vibration of CH bonds in the methylene (CH_3_) groups of PLA, and bands around 1180 cm^−1^, corresponding to the stretching vibration of CO bonds [24,25,26,27]. After 30, 180, and 300 days of fiber exposure to field conditions, minimal changes were observed in these characteristic bands, which were suggestive of minor alterations in the chemical structure but not proof of mulch degradation.

**Figure 9:**
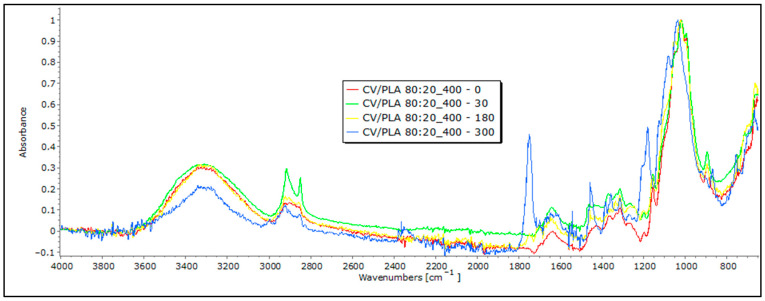
FTIR spectra of CV/PLA 80:20 nonwoven mulches of 400 g m^−2^ exposed for 0, 30, 180, and 300 days.

The characteristic band of cellulose-based viscose fibers, ranging from 3600 to 3200 cm^−1^, results from the stretching vibrations of hydroxyl (OH) groups. Bands in the 3000 to 2800 cm^−1^ range correspond to the carbon–hydrogen (CH) bond stretching vibrations in the aliphatic side chains of cellulose molecules, while the range from 1000 to 1200 cm^−1^ is indicative of CO bond stretching vibrations. Bands in the 1470–1432 cm^−1^ range are often associated with the bending vibrations of CH_2_ and CH_3_ groups in cellulose. Additionally, the band around 1735 cm^−1^ is linked to the CO stretching vibration of acetyl groups (hemiacetals) in cellulose. Although these bands are typical for viscose fibers, those in the 2400–1200 cm^−1^ range exhibit behavior that is more aligned with PLA fibers, suggesting a blend of the two. The band at 1750 cm^−1^ corresponds to the stretching vibration of the carbonyl group (CO) in the ester linkage of PLA. The presence of these bands collectively suggests a combination of cellulose-based viscose fibers and PLA fibers in the nonwoven mulch (Figure 9). These results point to the heterogeneity of the samples, and the degradation of the blends itself is difficult to monitor. Taking into account the obtained results, it can be concluded that these materials contain all of the mentioned (and necessary) bands, and changes in their intensity (for example, bands between 3600 and 3200 cm^−1^) may suggest the potential beginning of degradation. However, in most of the other characteristic bands and their intensity, there is no significant change, and it cannot be said with certainty whether degradation has occurred.

**Figure 10:**
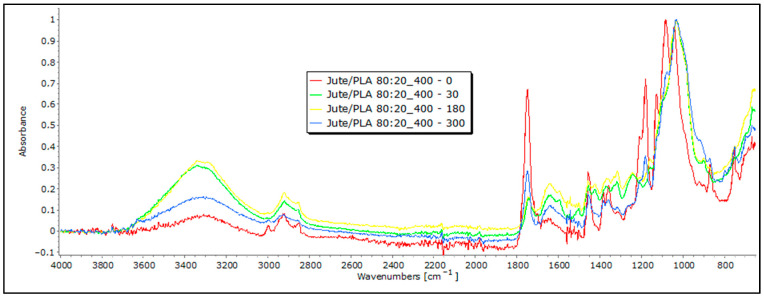
FTIR spectra of jute/PLA nonwoven mulches of 400 g m^−2^ exposed for 0, 30, 180, and 300 days.

**Figure 11:**
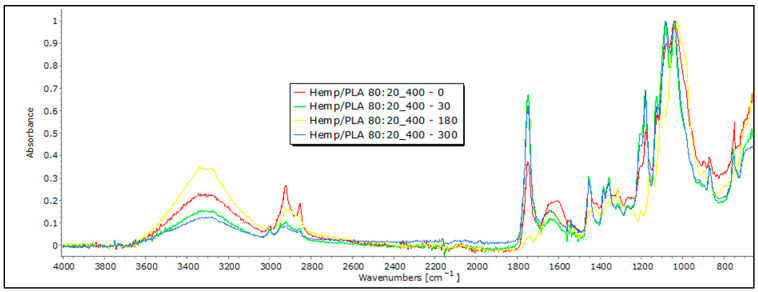
FTIR spectra of hemp/PLA nonwoven mulches of 400 g m^−2^ exposed for 0, 30, 180, and 300 days.

In blends of bast fibers with PLA, characteristic bands for cellulose in the region of 3600 to 3200 cm^−1^ are visible, representing OH stretching, CH stretching (around 2900 cm^−1^), CH_2_ bending (around 1200 cm^−1^), and CO stretching in the 1200–1000 cm^−1^ range. The characteristic bands for PLA are also visible at around 2950 cm^−1^, representing CH stretching, followed by 1750 cm^−1^, corresponding to the stretching of the CO group and –CH– groups around 1400 cm^−1^ for both samples: jute and hemp blends with PLA (Figure 10 and Figure 11). Again, these characteristic peaks indicate a homogenized sample for both the jute/PLA and hemp/PLA samples. The bands at 1748 cm^−1^, 1651 cm^−1^, and 1456 cm^−1^ indicate characteristic vibrations in the jute and PLA blend with the 0 sample of jute/PLA. Some bands, such as those around 3341 cm^−1^, 2919 cm^−1^, and 1745 cm^−1^, for the jute/PLA sample after 30 days, exhibit changes in absorbance compared to the 0 sample, explaining the increase in the tensile properties of the material. This trend of increased absorption due to increasing days of exposure of the mulch to field influences continues for the samples exposed for 180 and 300 days, confirming the changes caused by the exposure that led to an increase in the braking force. The hemp/PLA nonwoven mulch had more expressed bands, indicating the presence of lignin or other aromatic compounds, namely bands at 1748 cm^−1^, 1604 cm^−1^, and 1451 cm^−1^, rather than the jute/PLA samples. The bands at 1358 cm^−1^ and 1178 cm^−1^ indicate features of cellulose and hemicellulose in hemp. The absorbance values also changed for several bands in the hemp/PLA sample after 30 days of exposure. For instance, the peak at 1749 cm^−1^ (possibly from lignin) increased in intensity, suggesting that more lignin contributed to the higher breaking force of the material compared to the control sample. The peak at 1084 cm^−1^ (PLA) remains intense, suggesting that PLA is relatively stable in the initial stages of exposure. As already mentioned before, the FTIR analysis confirms the presence of both fibers, and thus, a coherent blend between the two fibers. Judging by the results and their intensity in the FTIR analysis, it cannot be claimed that any degradation of the samples occurred. In addition, the presence of impurities on the material prevented an accurate reading and conclusions regarding the influence of the field conditions on the material.

**Figure 12:**
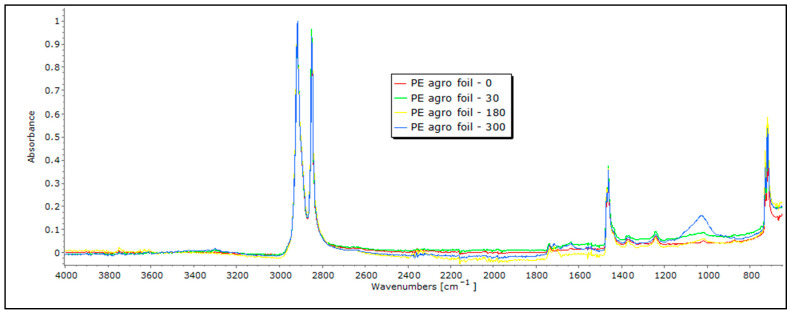
FTIR spectra of PE agro foil exposed for 0, 30, 180, and 300 days.

The primary distinguishing feature of PE (polyethylene) is the strong peak associated with the stretching vibration of its methylene (CH_2_) groups, typically in the 2916–2848 cm^−1^ range. Another characteristic peak corresponds to the bending vibrations of the CH_2_ groups, found around 1470–1465 cm^−1^. Additionally, PE contains CH_3_ groups, and their stretching vibrations result in a band at approximately 1375 cm^−1^. The bands associated with the stretching vibrations of carbon–carbon (CC) and carbon–hydrogen (CH) bonds within the PE polymer chain are situated within the range of 1200–1100 cm^−1^. These distinctive FTIR peaks in the PE agro foil spectrum arise from its polymer structure, which is composed primarily of long chains of carbon and hydrogen atoms [28,29]. An analysis of the FTIR spectrum of agro foil in this study indicates the absence of significant alterations in the FTIR peaks (Figure 12). This indicates that even after an exposure to environmental conditions of up to 300 days, no noticeable degradation in the PE agro foil has taken place, even though the mechanical tests suggest otherwise, namely a loss of up to 26% in the breaking force. 

This behavior is consistent with the well-known chemical stability of PE, making it highly resistant to various field conditions. Furthermore, the band at around 1050 cm^−1^ indicates the contamination of the material by the soil, which makes an accurate measurement impossible.

### 3.4. Soil Temperature and Moisture Beneath Mulches and Weed Suppression Capacity

From May to February, the highest recorded soil temperatures are mostly under agro foil, and they are higher than on the control fields and beneath all nonwoven mulches as a result of the raw material, structure, and color of the mulches. In June, the soil temperatures beneath cellulose and the cellulose/PLA blend nonwoven mulches are slightly higher than on the control field (for 2.0–3.1 °C), beneath agro foil (0.6 °C to 1.7 °C) and the PLA nonwoven mulches (for 1.2 °C to 2.3 °C) (Table 11).

From July to December, the soil temperatures beneath hemp and jute mulches, including nonwoven mulches produced by hemp and jute blends with PLA fibers, were higher than those on the control field. The temperature beneath viscose and the viscose blend mulches are lower than that on the control field from July to February. Respectively, the soil temperatures beneath the viscose mulches and viscose/PLA fiber blends revealed the lowest soil temperatures in the experiment period, except in June, when the highest temperatures were recorded. 

To determine the statistical significance of the temperature under the different mulches and in the control field in each month during the period of mulch exposure, Duncan’s new multiple range test (MRT) was performed (Table 12, Table 13 and Table 14).

The Duncan analysis revealed visible differences in the soil temperature between the mulches, agro foil, and control field from 30 to 120 days of exposure. CV 100% and CV/PLA differ from foil and the control field at 30 days, CV/PLA differs from foil and the control field at 60 days, and CV 100%, PLA 100%, and CV/PLA show significant differences at 90 days. No significant differences were found between the control field and all mulches at 90 days.

There are differences between the mulches and PE agro foil from 30 to 90 days of exposure and no difference between the mulches and foil at 60, 180, 240, and 300 days of exposure. CV and PLA exhibit similarities in the soil temperature measurements at 30 days, 90 days, and 120 days, while the other mulches and agro foil differ from the CV and PLA mulch.

There are differences in the soil temperature between the mulch blends, PE agro foil, and control field from 30 to 120 days of exposure, and there are no differences between the groups from 180 to 300 days. From 30 to 60 days of exposure, the CV/PLA mulch shows differences regarding the PE agro foil, while there is no significant difference between the agro foil and other mulches in the same period. At 120 days of exposure, jute/PLA shows a difference compared to the foil.

In May, the highest soil moisture was recorded on the control field, followed by the soil moisture beneath agro foil and nonwoven mulches (Table 15). From June to October, the soil moisture was higher beneath the nonwoven mulches than on the control field and beneath the agro foil. From July to December, the lowest moisture was recorded beneath the agro foil. In the winter, from December to February, the soil moisture on the control field was the highest. It can be concluded that nonwoven mulches could maintain soil moisture in the spring–summer period, when soil moisture is crucial for planted plants to grow, and they are better than conventional agro foil. It is necessary to conduct an open field study to determine the influence of newly produced nonwoven mulches on the growth, yield, and nutrient content of certain plant species.

There are significant differences in the soil moisture between the mulches, PE agro foil, and control field in almost all days of exposure, except for 120 days (Table 16). The most frequent difference between foil and the control field is shown by the CV/PLA mulch (30, 180, and 240 days), while mulches such as CV 100%, hemp 100%, and hemp/PLA also differ from agro foil and the control field at 180 days of exposure, and at 90 days of exposure, all mulches differ from the agro foil and the control field in terms of soil moisture.

There are significant differences in the soil moisture between the PE agro foil and 100% mulches at all days, except for 60 and 300 days of exposure, where there are no differences between those groups (Table 17). The PLA 100% mulch differs from agro foil at 30 days of exposure; from all mulches at 90 days; from hemp 100% mulch at 180 days; and finally, at 240 days of exposure, all mulches differ from agro foil in terms of the soil moisture except for the PLA 100% mulch.

There are differences only at 30, 180, and 240 days of exposure, while for other days, there are no significant differences in the soil moisture between PE agro foil and mulch blends (Table 18). At 30 days of exposure, the CV/PLA mulch differs from PE agro foil; at 180 days, CV/PLA and hemp/PLA mulch differ; and lastly, at 240 days, all mulches have significant differences in soil the moisture regarding agro foil.

Mulch produced by hemp fibers has the lowest suppression ability to weeds, although the percentage is small in the first six months of the field test (up to 14.3%) (Table 19). In the first six months of testing, mulch produced by the blend of hemp/PLA fibers and jute fibers allowed for up to 2.2% of weeds to pass through the mulches. In the sixth month of testing, an insignificant amount of weeds (up to 2.0%) passed through all mulches. In the eighth month of the field test, the highest percentage of weed passed through hemp mulch (34.3%), after which the mulch degraded. Although the jute mulch was degraded two months earlier, the weeds percentage that passed through the mulch was significantly lower (up to 2.2%). It can be concluded that all mulches have good suppression ability, whereas the suppression ability of hemp mulches decreases after six months of exposure to field conditions. The 20% of PLA fibers in the viscose, jute, and hemp mulch blends improved the weed control ability.

## 4. Conclusions

The mulches made from stem fibers, jute, and hemp completely decomposed after 10 months of field exposure. The jute mulches degraded first, in a period between 6 and 8 months, and the hemp mulches degraded after eight months. When observing the physical–mechanical properties of the nonwoven mulches over time, it is evident that the properties do not change linearly as expected. The reason for this could be due to changes in the structure of mulches during field exposure, i.e., the shrinkage and expansion of nonwoven fabrics. 

Microscopic images of viscose mulches show that the fibers have retained their shape and length but turned yellow due to a certain degree of decomposition. A significant change in the structure of the viscose mulches is also visible, i.e., the fibers have come closer to each other, causing a significant reduction in the thickness (47%) and an increase in density (19.3%). The breaking force decreased by only 10% in the MD and 27% in the CD after ten months of field exposure, while the FTIR spectral analyses provided compelling evidence of the chemical transformations occurring in viscose fibers due to the degradation process. 

The jute and hemp mulches degraded to such an extent that it was impossible to collect the mulches from the field, that is, pieces of the sample remained in the hands. A microscopy of the remains of jute and hemp nonwoven mulches shows the decomposition of jute and hemp fibers into elementary fibers. The reduction in the density of jute mulch before decomposition is 27.0%, and that of hemp is 36.1%. On the other hand, in the nonwoven mulch blends from jute and hemp with PLA fibers, the maintenance of the structure due to non-degraded PLA fibers is visible. The results revealed that the PLA fibers in mulch blends with jute, hemp, and viscose fibers slowed down the loss of mass per unit area and thickness and increased the air permeability. Also, this is additionally supported by the nonwoven mulches’ tensile properties. By comparing the breaking force and elongation at break of nonwoven jute and hemp mulches and their blends with PLA fibers, a less intensive decline until the end of the exposure time is evident. The viscose nonwoven mulch blend recorded a significant increase in the breaking force after two months, after which an alternating increase and decrease were recorded. After ten months, the breaking force was 180% higher in the MD and 73% higher in the CD. The dimensional changes in the mulches produced by viscose fibers and a mulch blend of CV/PLA fibers were the same, as they reduced by 6.3% in the MD and 0.7% in the CD. The addition of PLA fibers in the blend with viscose fibers contributed to the breaking force increase and the significantly smaller changes in the elongation at break in both directions after ten months of exposure. The FTIR analyses of cellulose nonwoven mulch blends with PLA fibers confirm the presence of both fibers (viscose and PLA, jute and PLA, and hemp and PLA), verifying a coherent blend between the two fibers, but the results and their intensity cannot confirm that there was degradation in the mulches. Additionally, the presence of impurities on the material prevented accurate reading and conclusions regarding the influence of filed conditions on the mulches. After ten months of field exposure, the nonwoven mulches made of PLA fibers did not show a change in the properties that indicate degradation. The FTIR analyses of the PLA mulches revealed minimal changes in the characteristic bands, suggesting minor alterations in the chemical structure without confirmation of mulch degradation.

The differences in the soil temperature and moisture between the mulches, agro foil, and control fields during exposure to the field conditions were recorded. The nonwoven mulches could maintain soil moisture in the spring–summer period, when soil moisture is crucial for planted plants to grow, and they are better than conventional agro foil, but it is necessary to perform an open field study to determine the influence of newly produced nonwoven mulches on the growth, yield, and nutrient content of certain plant species.

Newly produced mulches have good suppression ability, whereas the suppression ability of hemp mulches decreases after six months of exposure to field conditions. The addition of 20% PLA fibers in the viscose, jute, and hemp mulch blends improved the weed control ability. 

This investigation reveals the potential of replacing conventional agro foil with nonwoven mulches produced from jute, hemp, and viscose fibers, where the addition of 20% of PLA fibers prolongate the degradation time and contributes to maintaining the structure of the produced mulch blends.

## Figures and Tables

**Table 1:** The nonwoven mulch labels.

Label	Raw Material Composition
CV 100	100% viscose fibers
CV/PLA 80:20	80% viscose fibers, 20% PLA fibers
Jute 100	100% jute fibers
Jute/PLA 80:20	80% jute fibers, 20% PLA fibers
Hemp 100	100% hemp fibers
Hemp/PLA 80:20	80% hemp fibers, 20% PLA fibers
PLA 100	100% PLA fibers
Agro foil	conventional PE (polyethylene) agro foil

**Table 2:** Change in mass per unit area, thickness, and air permeability after ten months of field exposure compared to unexposed mulches.

Samples	Mass per Unit Area, %	Thickness, %	Air Permeability, %
CV 100	−23	−47	+28
CV/PLA 80:20	−18	−19	+23
Jute 100	D	D	D
Jute/PLA 80:20	−31	−2	+19
Hemp 100	D	D	D
Hemp/PLA 80:20	−33	−4	+25
PLA 100	0	+8	+2
Agro foil	+5	+25	impermeable

D denotes complete degradation.

**Table 3:** The breaking forces of mulches in machine directions tested during a period of field exposure and percentage breaking force change after ten months of exposure.

	Breaking Force in MD, N						Breaking Force Change, %
Months	0	2	4	6	8	10	0–10
CV 100	18.66	86.70	85.50	57.96	46.54	16.80	−10
SE	0.70	5.28	3.49	9.34	3.58	7.04	
CV/PLA 80:20	46.56	235.56	136.8	146.14	125.54	130.34	+180
SE	1.17	3.94	2.82	6.63	12.03	13.12	
Hemp 100	79.10	114.40	90.32	52.24	30.65	D	D
SE	5.55	17.66	9.10	9.93	1.61	D	
Hemp/PLA 80:20	197.24	114.38	90.32	268.48	204.48	196.00	−1
SE	7.10	17.67	9.10	18.24	19.53	24.66	
Jute 100	29.90	61.20	45.50	39.78	D	D	D
SE	0.54	4.34	4.43	5.46	D	D	
Jute/PLA 80:20	145.32	242.60	202.80	191.38	101.04	117.52	−19
SE	6.97	15.26	9.98	14.50	9.32	6.21	
PLA 100	83.16	330.20	301.00	281.58	295.04	304.94	+267
SE	3.82	6.41	8.84	5.55	14.44	8.31	
Agro foil	80.22	85.92	88.70	84.38	80.76	76.12	−5
SE	1.22	1.26	0.64	0.56	2.13	1.60	

SE denotes standard error (N), D denotes complete degradation, + denotes increase, and − denotes decrease.

**Table 4:** The breaking force of mulches in cross-machine directions tested during a period of field exposure and percentage breaking force change after ten months of exposure.

	Breaking Force in CD, N						Breaking Force Change, %
Months	0	2	4	6	8	10	0–10
CV 100	26.24	130.20	126.90	57.98	56.08	19.10	−27
SE	1.60	4.92	8.45	11.73	8.59	4.49	
CV/PLA 80:20	79.52	264.78	184.00	151.90	132.24	137.38	+73
SE	4.29	8.22	4.95	9.21	6.68	7.44	
Hemp 100	96.20	152.40	111.20	98.00	77.40	D	D
SE	8.45	20.53	10.17	11.65	8.92	D	
Hemp/PLA 80:20	389.22	152.44	111.20	319.54	210.42	216.72	−44
SE	26.22	20.52	10.17	7.25	11.08	8.87	
Jute 100	42.50	66.40	55.60	49.28	D	D	D
SE	2.55	4.65	8.41	6.47	D	D	
Jute/PLA 80:20	160.54	286.60	235.00	193.08	150.08	165.50	+3
SE	7.40	13.37	12.65	12.98	6.03	8.98	
PLA 100	96.88	446.80	382.20	444.76	406.34	410.08	+323
SE	1.79	1.43	5.48	2.29	4.87	7.00	
Agro foil	133.34	126.92	112.30	104.74	89.48	99.16	−26
SE	1.79	1.43	5.48	2.29	4.87	7.00	

SE denotes standard error (N), D denotes complete degradation, + denotes increase, and − denotes decrease.

**Table 5:** The statistical analysis of the mulches’ breaking force in the MD and CD.

Samples Tested	*p*-Value
The breaking force of all nonwoven mulches in MD (CV, jute, hemp, PLA, CV/PLA, jute/PLA, hemp/PLA)	4.09 × 10^−10^
The breaking force of nonwoven mulch blends in MD (CV/PLA, jute/PLA, hemp/PLA)	0.90
The breaking force of 100% nonwoven mulches in MD (CV, jute, hemp, PLA)	1.22 × 10^−9^
The breaking force of nonwoven mulch blends and agro foil in MD (CV/PLA, jute/PLA, hemp/PLA, agro foil)	0.01
The breaking force of 100% nonwoven mulches and agro foil in MD (CV, jute, hemp, PLA, agro foil)	1.56 × 10^−11^
The breaking force of all nonwoven mulches in CD (CV, jute, hemp, PLA, CV/PLA, jute/PLA, hemp/PLA)	3.94 × 10^−11^
The breaking force of nonwoven mulch blends in CD (CV/PLA, jute/PLA, hemp/PLA)	0.81
The breaking force of 100% nonwoven mulches in CD (CV, jute, hemp, PLA)	2.29 × 10^−9^
The breaking force of nonwoven mulch blends and agro foil in CD (CV/PLA, jute/PLA, hemp/PLA, agro foil)	0.04
The breaking force of nonwoven mulches and agro foil in CD (CV, jute, hemp, PLA, agro foil)	4.03 × 10^−11^

**Table 6:** Elongation at break of mulches in machine direction, tested during a period of field exposure and elongation force change in percentage after ten months of exposure.

	Elongation at Break in MD, %						Change in Elongation at Break, %
Months	0	2	4	6	8	10	0–10
CV 100	22.27	29.16	19.92	4.68	6.91	4.89	−78.04
SE	0.93	2.30	0.88	0.15	1.11	0.84	
CV/PLA 80:20	37.89	50.73	35.85	47.99	16.36	31.15	−17.79
SE	0.91	1.42	2.33	2.76	1.91	3.31	
Hemp 100	40.85	28.76	22.03	10.60	6.10	D	D
SE	1.55	1.39	1.70	2.20	0.05	D	
Hemp/PLA 80:20	90.38	28.76	22.03	64.30	38.64	59.96	−33.66
SE	2.67	1.39	1.70	2.72	3.54	6.42	
Jute 100	21.99	20.40	16.74	15.34	D	D	D
SE	0.88	0.95	1.41	1.50	D	D	
Jute/PLA 80:20	71.91	51.11	33.51	31.61	24.99	27.08	−62.35
SE	3.33	3.03	1.51	2.41	4.16	0.88	
PLA 100	82.78	88.55	79.61	70.71	75.81	75.23	−9.12
SE	4.18	1.87	1.53	1.37	1.91	3.82	
Agro Foil	197.80	7.35	6.00	8.37	5.91	5.91	−97.01
SE	11.62	0.28	0.10	0.74	0.49	0.51	

SE denotes the standard error (N), D denotes complete degradation, + denotes increase, and − denotes decrease.

**Table 7:** Elongation at break of mulches in cross-machine directions tested during a period of field exposure and elongation force change in percentage after ten months of exposure.

	Elongation at Break in CD, %						Change in Elongation at Break, %
Months	0	2	4	6	8	10	0–10
CV 100	15.13	12.20	8.29	6.94	4.65	4.00	−73.56
SE	1.39	0.63	0.42	0.54	0.95	0.07	
CV/PLA 80:20	34.02	33.64	25.77	23.15	29.89	22.24	−34.64
SE	1.19	2.49	1.32	0.93	2.29	2.81	
Hemp 100	20.84	18.57	13.15	14.89	6.23	D	D
SE	1.77	1.68	1.17	1.08	0.65	D	
Hemp/PLA 80:20	49.15	18.57	13.15	38.55	72.91	31.85	−35.21
SE	2.36	1.68	1.17	2.18	4.04	2.35	
Jute 100	12.44	13.37	11.09	11.09	D	D	D
SE	0.68	0.41	1.02	1.12	D	D	
Jute/PLA 80:20	50.14	33.40	47.65	48.82	55.72	53.54	+6.78
SE	1.46	1.43	3.30	1.93	1.70	1.72	
PLA 100	55.92	69.22	65.95	66.01	66.08	61.74	+10.42
SE	0.84	0.61	0.74	0.54	2.67	1.94	
Agro Foil	95.33	81.70	59.32	49.74	36.15	43.53	−54.34
SE	7.06	3.93	6.58	2.19	6.73	8.00	

SE denotes standard error (N), D denotes complete degradation, + denotes increase, and − denotes decrease.

**Table 8:** The statistical analysis of the mulches’ elongation at break in the MD and CD.

Samples Tested	*p*-Value
The elongation at break of all nonwoven mulches in MD (CV, jute, hemp, PLA, CV/PLA, jute/PLA, hemp/PLA)	1.94 × 10^−3^
The elongation at break of nonwoven mulches blends in MD (CV/PLA, jute/PLA, hemp/PLA)	0.79
The elongation at break of 100% nonwoven mulches in MD (CV, jute, hemp, PLA)	2.30 × 10^−10^
The elongation at break of nonwoven mulch blends and agro foil in MD (CV/PLA, jute/PLA, hemp/PLA, agro foil)	0.99
The elongation at break of 100% nonwoven mulches and agro foil in MD (CV, jute, hemp, PLA, agro foil)	3.73 × 10^−3^
The elongation at break of all nonwoven mulches in CD (CV, jute, hemp, PLA, CV/PLA, jute/PLA, hemp/PLA)	4.21 × 10^−16^
The elongation at break of nonwoven mulches blends in CD (CV/PLA, jute/PLA, hemp/PLA)	6.37 × 10^−3^
The elongation at break of 100% nonwoven mulches in CD (CV, jute, hemp, PLA)	1.10 × 10^−17^
The elongation at break of nonwoven mulches blends and agro foil in CD (CV/PLA, jute/PLA, hemp/PLA, agro foil)	4.0 × 10^−4^
The elongation at break of nonwoven mulches and agro foil in CD (CV, jute, hemp, PLA, agro foil)	4.22 × 10^−14^

**Table 9:** Dimensional changes in mulches (cm) in MD and CD during a period of filed exposure and dimensional change in percentage after ten months of exposure.

Period of Exposure	0	0–6 Months		6–8 Months		8–10 Months		In Total, %	
Sample	MD/CD	MD	CD	MD	CD	MD	CD	MD	CD
CV 100	150.0	145.0	155.0	155.3	135.0	140.5	149.0	−6.3	−0.7
CV/PLA 80:20	150.0	141.0	149.0	132.4	150.1	140.5	149.0	−6.3	−0.7
Hemp 100	150.0	157.0	146.0	D	D	D	D	D	D
Hemp/PLA 80:20	150.0	144.5	147.0	144.9	153.3	152.2	143.0	1.5	−4.7
Jute 100	150.0	151.0	148.5	D	D	D	D	D	D
Jute/PLA 80:20	150.0	143.0	151.0	153.2	142.5	139.0	149.0	−7.3	−0.7
PLA 100	150.0	147.5	152.5	149.3	150.5	151.5	152.3	1.0	1.5
Agro foil	150.0	145.0	155.0	155.3	135.0	140.5	149.0	−6.3	−0.7

D denotes complete degradation, + denotes increase, and − denotes decrease.

**Table 10:** The density of mulches during field exposure and change in density (%).

	Time of Exposure to Field Conditions, Months						Change in Density, %
Months	0	2	4	6	8	10	
CV 100	61.7	88.4	85.0	87.3	87.2	73.6	+19.3
CV/PLA 80:20	84.9	93.1	90.1	87.9	91.7	85.2	+0.4
Hemp 100	86.3	60.4	60.9	63.0	D	D	−27.0
Hemp/PLA 80:20	91.9	73.1	68.4	62.0	67.0	71.8	−21.9
Jute 100	108.8	75.8	66.8	96.8	69.5	D	−36.1
Jute/PLA 80:20	98.2	79.4	81.2	81.0	77.4	76.7	−21.9
PLA 100	65.1	58.5	52.5	61.6	58.4	60.3	−7.4
Agro foil	335.7	436.1	426.2	170.9	197.3	266.2	−20.7

**Table 11:** The average monthly temperature of soil on the control field and beneath mulches in the period from May 2022 to February 2023.

Year	2022								2023	
Month	May	June	July	Aug	Sept	Oct	Nov	Dec	Jan	Feb
Air Temperature, °C	17.7	22.4	22.9	22.4	15.9	13.0	7.2	3.9	3.7	3.3
CV 100, °C	16.0	23.5	20.2	19.9	16.8	14.0	11.8	4.7	4.2	3.2
CV/PLA 80:20, °C	16.4	23.1	20.1	19.8	16.8	14.0	11.8	4.7	4.2	3.5
Hemp 100, °C	17.9	22.6	21.5	20.7	17.3	14.3	16.3	5.1	4.5	3.7
Hemp/PLA 80:20, °C	17.6	22.4	21.5	20.6	17.2	14.3	12.4	5.1	4.4	3.9
Jute 100, °C	17.7	23.3	21.7	20.6	17.2	14.1	12.0	4.9	4.4	3.7
Jute/PLA 80:20, °C	17.8	23.3	21.6	20.6	17.4	14.4	12.5	5.1	4.3	3.7
PLA 100, °C	18.3	21.2	20.5	20.3	17.4	14.4	12.7	5.4	4.6	3.8
Agro foil, °C	18.8	21.8	22.0	20.9	17.6	14.6	12.6	5.1	4.7	4.0

**Table 12:** Duncan statistical analysis of soil temperature beneath the 100% mulches and mulch blends with PE agro foil and control field.

Samples	30	60	90	120	180	240	300
CV 100%	16.04 c	21.59 a	20.57 c	19.92 de	16.11 a	8.60 a	3.71 a
Jute 100%	17.70 ab	22.06 a	22.13 a	20.58 abc	16.41 a	8.78 a	4.04 a
Hemp 100%	17.87 ab	21.95 a	22.01 ab	20.68 ab	16.55 a	10.51 a	4.06 a
PLA 100%	18.28 a	20.56 ab	21.00 bc	20.33 bcd	16.60 a	9.32 a	4.18 a
CV/PLA 80:20	16.39 bc	19.55 b	20.52 c	19.65 e	16.18 a	8.58 a	3.88 a
Jute/PLA 80:20	17.80 ab	20.89 ab	22.12 a	20.61 abc	16.68 a	9.04 a	4.01 a
Hemp/PLA 80:20	17.64 ab	20.54 ab	21.92 ab	20.58 abc	16.54 a	9.02 a	4.14 a
PE Agro foil	18.76 a	21.64 a	22.46 a	21.03 a	16.85 a	9.14 a	4.36 a

Where a, b, c, d, e represent groupings of means. Groups that share a letter are not significantly different from each other, while groups with different letters are significantly different. The ordering of the letters reflects the magnitude of the means, with groups that are more similar having the same letter or sequence of letters.

**Table 13:** Duncan statistical analysis of soil temperature beneath the 100% mulches and PE agro foil.

Samples	30	60	90	120	180	240	300
CV 100%	16.04 b	21.59 a	20.38 b	19.92 c	16.11 a	8.60 a	3.71 a
Jute 100%	17.00 a	22.06 a	22.01 a	20.58 ab	16.41 a	8.78 a	4.04 a
Hemp 100%	17.87 a	21.95 a	21.89 a	20.68 ab	16.55 a	10.51 a	4.06 a
PLA 100%	18.28 a	20.56 a	20.82 b	20.33 bc	16.60 a	9.32 a	4.18 a
PE Agro foil	18.76 a	21.64 a	22.33 a	21.03 a	16.85 a	9.14 a	4.36 a

Where a, b, c represent groupings of means. Groups that share a letter are not significantly different from each other, while groups with different letters are significantly different. The ordering of the letters reflects the magnitude of the means, with groups that are more similar having the same letter or sequence of letters.

**Table 14:** Duncan statistical analysis of soil temperature beneath the mulch blends and PE agro foil.

Samples	30	60	90	120	180	240	300
CV/PLA 80:20	16.39 b	19.55 b	20.36 b	19.65 c	16.18 a	8.58 a	3.88 a
Jute/PLA 80:20	17.80 ab	20.89 a	22.01 a	20.61 a	16.68 a	9.04 a	4.01 a
Hemp/PLA 80:20	17.64 ab	20.54 ab	21.75 a	20.58 ab	16.54 a	9.02 a	4.14 a
PE Agro foil	18.76 a	21.64 a	22.33 a	21.03 bc	16.85 a	9.14 a	4.36 a

Where a, b, c represent groupings of means. Groups that share a letter are not significantly different from each other, while groups with different letters are significantly different. The ordering of the letters reflects the magnitude of the means, with groups that are more similar having the same letter or sequence of letters.

**Table 15:** The average relative moisture of soil on the control field and beneath mulches in the period from May 2022 to February 2023.

Year	2022								2023	
Month	May	June	July	Aug	Sept	Oct	Nov	Dec	Jan	Feb
RH, %	93.0	90.0	90.0	91.0	94.0	94.0	96.0	97.0	97.0	96.0
Precipitation, %	53.9	52.0	69.0	22.2	280.0	27.9	119.5	132.3	171.0	27.6
CV 100, °C	24.1	23.5	17.6	16.8	19.3	23.7	24.3	22.7	20.9	19.9
CV/PLA 80:20, °C	22.7	23.1	17.4	16.2	19.7	24.3	24.6	23.7	22.1	20.4
Hemp 100, °C	23.4	22.6	18.2	15.9	19.4	24.0	23.9	22.5	21.2	18.6
Hemp/PLA 80:20, °C	24.5	22.4	16.9	15.3	19.8	24.1	23.7	23.5	22.2	19.6
Jute 100, °C	23.8	23.3	17.7	15.3	19.0	23.2	23.6	21.9	20.7	17.3
Jute/PLA 80:20, °C	23.6	23.3	17.2	15.0	18.7	23.0	23.3	22.9	21.3	18.7
PLA 100, °C	23.1	21.2	15.5	16.7	18.1	22.6	20.9	21.4	22.4	21.2
Agro foil, °C	24.7	21.8	12.7	12.1	16.2	20.3	19.9	21.0	21.2	19.6
Control field	25.1	20.4	13.8	14.5	17.2	20.6	22.1	23.2	24.6	21.8

RH is the average relative humidity of air, %.

**Table 16:** Duncan statistical analysis of soil moisture beneath the 100% mulches and mulch blends with PE agro foil and control field.

Samples	30	60	90	120	180	240	300
CV 100%	24.14 abcd	24.60 a	17.90 a	17.44 a	20.19 a	23.61 ab	20.45 bc
Jute 100%	23.83 abcd	24.09 a	18.20 a	15.78 a	19.76 ab	22.82 b	19.01 c
Hemp 100%	23.44 bcd	23.56 ab	18.26 a	16.24 a	20.61 a	23.23 ab	19.90 bc
PLA 100%	23.11 cd	22.24 b	15.83 b	17.75 a	19.31 ab	21.28 c	21.79 ab
CV/PLA 80:20	22.72 d	23.86 a	18.03 a	16.67 a	20.57 a	24.32 a	21.26 abc
Jute/PLA 80:20	23.59 bcd	24.28 a	17.63 ab	15.37 a	19.59 ab	23.61 ab	19.98 bc
Hemp/PLA 80:20	24.48 abc	23.39 ab	17.20 ab	15.50 a	20.71 a	23.68 ab	20.91 abc
PE Agro foil	24.71 ab	23.34 ab	13.32 c	12.82 a	16.95 b	20.54 c	20.43 bc
Control field	25.17 a	22.18 b	13.73 c	15.57 a	17.66 ab	22.54 b	23.19 a

Where a, b, c, d represent groupings of means. Groups that share a letter are not significantly different from each other, while groups with different letters are significantly different. The ordering of the letters reflects the magnitude of the means, with groups that are more similar having the same letter or sequence of letters.

**Table 17:** Duncan statistical analysis of soil moisture beneath the 100% mulches and PE agro foil.

Samples	30	60	90	120	180	240	300
CV 100%	24.13 ab	24.59 a	17.89 a	17.44 a	20.19 a	23.58 a	20.45 a
Jute 100%	23.82 ab	24.08 a	18.20 a	15.77 a	19.76 ab	22.82 a	19.09 a
Hemp 100%	23.43 ab	23.54 a	18.25 a	16.23 a	20.61 a	23.19 a	19.90 a
PLA 100%	23.10 c	22.23 b	15.81 b	17.76 a	19.31 ab	21.28 b	21.79 a
PE Agro foil	24.70 a	23.33 ab	13.30 c	12.80 a	16.95 b	20.53 b	20.43 a

Where a, b, c represent groupings of means. Groups that share a letter are not significantly different from each other, while groups with different letters are significantly different. The ordering of the letters reflects the magnitude of the means, with groups that are more similar having the same letter or sequence of letters.

**Table 18:** Duncan statistical analysis of soil moisture beneath the mulch blends and PE agro foil.

Samples	30	60	90	120	180	240	300
CV/PLA 80:20	22.72 b	23.85 a	18.02 a	16.65 a	20.57 a	24.31 a	21.24 a
Jute/PLA 80:20	23.59 ab	24.28 a	17.61 a	15.35 a	19.59 ab	23.23 b	19.96 a
Hemp/PLA 80:20	24.46 a	23.37 a	17.19 a	15.47 a	20.71 a	23.69 ab	20.91 a
PE Agro foil	24.70 a	23.33 a	13.30 a	12.80 a	16.95 b	20.53 c	20.43 a

Where a, b, c represent groupings of means. Groups that share a letter are not significantly different from each other, while groups with different letters are significantly different. The ordering of the letters reflects the magnitude of the means, with groups that are more similar having the same letter or sequence of letters.

**Table 19:** The weediness on the control field and beneath mulches in the period from May 2022 to February 2023.

Year	2022					2023
Month	June	July	Aug	Oct	Dec	Feb
CV 100, °C	-	-	-	2.0	10.3	-
CV/PLA 80:20, °C	-	-	-	0.1	2.6	1.4
Hemp 100, °C	1.6	12.4	14.3	2.0	34.3	D
Hemp/PLA 80:20, °C	0.1	-	-	0.2	2.8	1.49
Jute 100, °C	0.5	1.7	2.2	1.6	D	D
Jute/PLA 80:20, °C	-	-	-	0.9	3.1	2.9
PLA 100, °C	-	-	-	1.0	-	-
Agro foil, °C	-	-	-	0.2	1.2	-
Control field	100	100	100	100	100	100

D denotes complete degradation.

## Data Availability

All data are contained within the article.
